# Maternal Body Mass Index and Risk of Obstetric Anal Sphincter Injury

**DOI:** 10.1155/2014/395803

**Published:** 2014-04-15

**Authors:** Marie Blomberg

**Affiliations:** Department of Obstetrics and Gynaecology and Department of Clinical and Experimental Medicine, Linköping University, 581 85 Linköping, Sweden

## Abstract

*Objective*. To estimate the association between maternal obesity and risk of three different degrees of severity of obstetric anal sphincter injury. *Methods*. The study population consisted of 436,482 primiparous women with singleton term vaginal cephalic births between 1998 and 2011 identified in the Swedish Medical Birth Registry. Women were grouped into six categories of BMI. BMI 18.5–24.9 was set as reference. Primary outcome was third-degree perineal laceration, partial or total, and fourth-degree perineal laceration. Adjustments were made for year of delivery, maternal age, fetal head position at delivery, infant birth weight and instrumental delivery. *Results*. The overall prevalence of third- or four-degree anal sphincter injury was 6.6% (partial anal sphincter injury 4.6%, total anal sphincter injury 1.2%, unclassified as either partial and total 0.2%, or fourth degree lacerations 0.6%). The risk for a partial, total, or a fourth-degree anal sphincter injury decreased with increasing maternal BMI most pronounced for total anal sphincter injury where the risk among morbidly obese women was half that of normal weight women, OR 0.47 95% CI 0.28–0.78. *Conclusion*. Obese women had a favourable outcome compared to normal weight women concerning serious pelvic floor damages at birth.

## 1. Introduction

Risk factors for obstetric anal sphincter injury are extensively studied and found to be primiparity, assisted delivery, persistent occipitoposterior position, high birth weight, and prolonged second stage of labor [1–4]. Maternal obesity is associated with a number of severe obstetric and neonatal outcomes including large for gestational age infants and prolonged labor [5, 6]. Whether maternal obesity itself is a risk factor for anal sphincter injury or perineal adipose tissue protects the anal sphincter is still unclear. Prior study results differ. Maternal weight and maternal body mass index (BMI) were shown to be significantly higher among women with third and fourth degree perineal tears compared to unaffected women [7] but, in large register studies, no association between maternal obesity and anal sphincter injuries was found [5, 8, 9]. There are also studies indicating that maternal obesity could be protective against anal sphincter lacerations overall [10, 11].

All studies, however, evaluated third- and fourth-degree anal sphincter injuries together and made no separation between whether the external anal sphincter is partly damaged or entirely disrupted which is of great clinical importance since the long-term outcome for the women depends on the degree of the laceration as well as the adequacy of the primary repair [12, 13].

The objective of the present study was to estimate, in a large data set from the Swedish Medical Birth Registry, the association between maternal obesity and risk of different degrees of obstetric anal sphincter injury among primiparous women with singleton term births after adjustment for late fetal head position at delivery, instrumental delivery, and infant birth weight, all well documented risk factors for anal sphincter injury. Secondly, another objective was to study the effect of late fetal head position, birth weight, and instrumental delivery on the the risk of anal sphincter injury in the six different maternal BMI classes.

## 2. Materials and Methods

The study population consisted of 436,482 primiparous women with singleton term (gestational week ≥ 37 + 0) vaginal cephalic births in Sweden from January 1, 1998, to December 31, 2011, with available information on fetal head position at delivery and infant birth weight. They were identified from the Swedish Medical Birth Registry. Medical and other data on almost all (99%) deliveries in Sweden are listed in the register. It is based on copies of the standardized medical record forms completed at the antenatal health care centers at the start of prenatal care, usually in gestational weeks 10–12, records from the delivery units, and records from the pediatric examination of the infant. Ninety percent of women presented themselves to the antenatal health care center during the first trimester of their pregnancy. The system is identical throughout the country. A description and validation of the register content is available [14].

Midwives measured maternal weight and height (if not known to the woman) and recorded values in a standardized form at the woman's first visit to the antenatal health care center. Body mass index in kg/m^2^ was then calculated from maternal weight and height data. Women were grouped into six categories of BMI: underweight (less than 18.5), normal weight (18.5–24.9), overweight (25–29.9), obese class I (30–34.9), obese class II (35–39.9), and obese class III (40 or more) according to World Health Organization (WHO) classification [15]. Obesity class III is equivalent to morbid obesity in this study.

The outcomes studied were registered in the Swedish Medical Birth Registry using the International Classification of Diseases and Related Health Problems, Tenth Edition (ICD-10). The ICD codes for each outcome were as follows:

- second degree perineal laceration during delivery is as follows: perineal laceration, rupture, or tear involving pelvic floor and/or perineal muscles and/or vaginal muscles (International Classification of Diseases, Tenth Edition [ICD-10] code O70.1);
- third degree perineal laceration during delivery is as follows: perineal laceration, rupture, or tear involving anal sphincter and/or rectovaginal septum (ICD-10 code O70.2); in the Swedish version of ICD-10, this group is divided into O70.2A which is partial anal sphincter injury and O70.2B which is total anal sphincter injury;
- fourth degree perineal laceration during delivery is as follows: perineal laceration, rupture, or tear as in O70.2 but also involving the anal/rectal mucosa (ICD-10 code O70.3).

Another available ICD 10 code in the register is O70.2X which means that the anal sphincter injury could not be classified into either partial or total. A total number of 1036 women had that ICD code and they were only included in overall analyses concerning women with third degree lacerations.

A total number of 65 (0.2%) women included in the study had two diagnoses of anal sphincter injury. The most common combination was O70.2B and O70.3 (26 women). These women were classified as O70.3. The second most common combination was O70.2 and O70.2A; these 14 women were classified as O70.2A. The third most common combination was O70.2A and O70.3 (11 women) and these women were classified as O70.3. The other women had varied combinations of diagnoses and the hierarchy used was that the most specified and serious diagnoses were chosen in favor of an unspecified and milder one.

Other obstetric factors studied in relation to perineal lacerations were year of birth (1998–2011), maternal age (seven 5-year classes), persistent occiput posterior position at delivery, instrumental delivery (vacuum and forceps), and infant birth weight (six classes, <3000 grams, 3000–3499, 3500–3999, 4000–4499, 4500–4999, and ≥5000). Information on these variables was also obtained from the Swedish Medical Birth Registry. In the present population of primiparous women with singleton term vaginal cephalic births, data on birth weight was missing in 739 cases (0.2%), data on fetal head position at delivery in 1567 cases (0.4%), and data without a reasonable maternal age in 1439 cases (0.3%). All individuals had data on year of birth and mode of delivery.

The effect of birth weight, instrumental delivery, and late fetal head position were also studied over the six maternal BMI strata.

Adjusted odds ratios (OR) were determined using Mantel-Haenszel technique [16]. Estimates of 95% confidence intervals (CI) were made with a test-based method [17], based on the Mantel-Haenszel chi-square test. OR for each variable studied in relation to perineal lacerations was adjusted for all other variables.

The Regional Ethical Committee in Linköping has approved the study.

## 3. Results

A total of 385961 (88.4%) women had available data in the register on both weight and height that enabled calculation of maternal BMI. The prevalence of obesity among primiparous women (BMI greater than 30) was 8.0% and the distribution in the three obesity classes was class I, 5.9%; class II, 1.6%; and class III, 0.5%.

The overall prevalence of third- or fourth-degree anal sphincter injury in this group of primiparous women was 6.6 (*n* = 28915)%. Partial anal sphincter injury occurred in 4.6% (*n* = 19923), total anal sphincter injury in 1.2% (*n* = 5456), and fourth degree lacerations in 0.6% (*n* = 2500). The number of unspecified anal sphincter injuries (not classified as partial or total) was 0.2% (*n* = 1036). The prevalence did not change substantially over the 14-year period studied (data not shown).

In Table 1, it is shown that the overall risk of getting an anal sphincter injury at delivery decreased significantly with increasing maternal BMI. ORs were adjusted for maternal age, fetal head position at delivery, mode of delivery, and infant birth weight. Compared to women with normal BMI there was a 30% increased risk for anal sphincter injury among underweight women. The OR among women with missing data on BMI was 0.96 (95% CI 0.93–1.00) indicating that this group was similar to women with available data on maternal weight and height.

The strongest risk factor for anal sphincter injury evaluated in this study was infant birth weight reaching an over 10-fold increased risk, if the infant weigh was above 5000 g, but notably the risk was significantly increased also in the more frequent group of infants weighing 4500–4999 g, OR 6.33 (95% CI 5.97–6.92). Older maternal age up to the age of 40 years seemed to be a risk factor for any anal sphincter injury compared to women aged 25–29. In the oldest maternal groups (above 40 years of age), the risk seemed to be decreased compared to the reference group (aged 25–29) although nonsignificant with wide confidence intervals. The lowest risk of anal sphincter injury was found among teenagers. Other identified risk factors for anal sphincter injury were occiput posterior position of the fetal head at delivery, OR 1.36 (95% CI 1.29–1.44), and instrumental delivery, OR 2.74 (95% CI 2.67–2.81). The absolute majority of instrumental deliveries in Sweden are performed by vacuum extraction.

**Figure 1:**
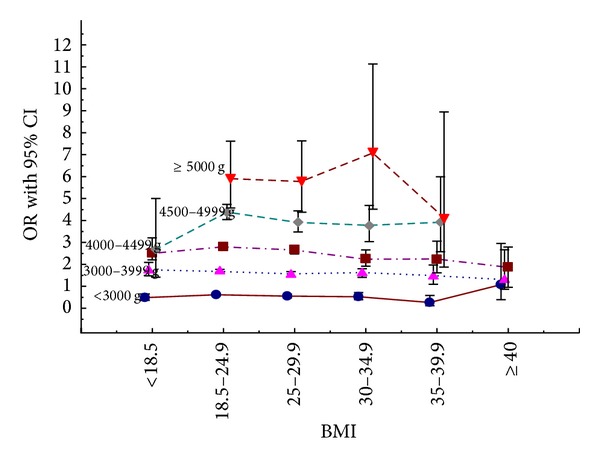
The effects of birth weight on the risk of any anal sphincter injury in the different maternal body mass index classes. Adjustments were made for year of birth, maternal age, instrumental delivery, and fetal head position. If there were less than five cases in a stratum including a specific maternal BMI class and a specific birth weight odds ratio was not calculated.

Table 2 presents risk factors for the three subgroups of anal sphincter injuries separately. Again ORs were estimated after adjustments for all other risk factors studied. The risk for a partial, total, or a fourth-degree anal sphincter injury decreased with increasing maternal BMI most pronounced for a total anal sphincter injury where the risk among morbidly obese women was half that of normal weight women. High infant birth weight (4500–4999 g) increased the risk markedly for total anal sphincter injury and fourth-degree laceration as well as infant birth weight above 5000 g but that group is quite small. Instrumental delivery increased the risk of all three subgroups equally but the OR for increasing degree of anal sphincter injury was increased with occiput posterior position of the fetal head at delivery.  Table 3 shows the effect of fetal head position at delivery as well as the effect of instrumental delivery at different maternal BMI. There was a significantly increased risk for any anal sphincter injury associated with instrumental delivery in all maternal BMI classes but the ORs decreased with increasing maternal BMI. The occiput posterior position of the fetal head at birth as a risk factor for any sphincter injury was increased in all maternal BMI classes although data must be interpreted with caution because of low numbers of this position among morbidly obese women. Figure 1 shows the effect of infant birth weight on the risk for any anal sphincter injury according to maternal BMI group. This is the most prominent risk factor of anal sphincter injury but, for infants weighing 3500 g–4500 g, the risk decreases with increasing BMI. The opposite was found for infants weighing 4500 g–4999 g where the ORs were highest in the morbidly obese group.

## 4. Discussion

This large population-based cohort study including primiparous women with term pregnancies showed that the risk of partial anal sphincter injury, total sphincter injury, and fourth-degree perineal laceration decreased with increasing maternal BMI. The overall risk for any anal sphincter injury among morbidly obese women was reduced with 25% compared to normal weight women. For total anal sphincter injury the risk in the morbidly obese group was half that of normal weight women. The strongest risk factor for anal sphincter injury evaluated in this study was the size of the infant. The effect of birth weight on the risk for anal sphincter injury slightly decreased with increasing maternal BMI giving that the risk for anal sphincter injury if the infant has a birth weight above 4000 g was lower among morbidly obese women compared to average weight women. A similar but less pronounced trend could be seen for instrumental delivery.

There are earlier studies, based on large register datasets and one meta-analysis, estimating the risk for anal sphincter injury over the maternal BMI strata and they showed no association between maternal obesity and anal sphincter injuries [5, 8, 9]. It could be due to adjustments not including risk factors associated with perineal tears. Lindholm and Altman presented a decreased risk for grade three and four anal sphincter lacerations together among women with BMI 30 to 34.9 as well as for women with BMI ≥ 35 compared to women with BMI < 25 [10]. Their prevalence rate of any anal sphincter injury was lower than in the present study but they did not restrict the study population to term infants. In another study based on electronic medical records maternal BMI at delivery reduced the risk for third or four degree laceration with 30% in the morbidly obese group which is in accordance with results in the present study. Interestingly, the prevalence of any sphincter laceration in the American dataset was equal to that in the present study despite the huge difference in cesarean section rates (43.8 compared to 16.4%) [11]. Recently reported rates of anal sphincter injuries among primiparous women in US and England, 5.8-5.9%, were slightly lower than in the present study. However there are contries presenting much lower rates of anal spincter injuries in the primiparous group, for example Finland where the rate was 1.7% in the year 2011 [11, 18, 19]. Observed variations in prevalence rates could be due to a number of factors, that is, different registration routines, different competence in the diagnosing of the injury, different episiotomy rates, and variations in use of manual perineal protection during delivery and differences in population characteristics. The knowledge about the three degrees of severity of anal sphincter injuries in relation to maternal BMI is sparse. The protective effect of morbid obesity seemed to be most pronounced for total anal sphincter injuries, reaching an almost 50% reduced risk.

The observed reduced risk of diagnosed anal sphincter injuries could be looked upon from different points of view. The first question would be if the observed reduced risk of anal sphincter injury among obese women is true or false? False in the sense that the decreased risk was due to lower detection rate of injuries in the obese group of women. It could be possible that the voluminous amount of fat tissue in the perineal region complicates adequate examination of the anatomy. There are studies indicating that adding ultrasound of the anal sphincter to the immediate examination after delivery increases detection rate of ruptured anal sphincters [20]. This tool may be more efficient in the obese group of women but the method is not yet to my knowledge evaluated over the BMI strata. Instrumental delivery increases the risk for anal sphincter injuries most pronounced for forceps [4]. Forceps is practically not used in Sweden and there was no major difference between the usages of vacuum extraction among normal weight women (7.5%) compared to obese women (7.0%). Instrumental delivery was also included as a confounder in the analysis. If the negative association between maternal obesity and risk of anal sphincter injuries is true, a clinical speculation could be that the increased amount of adipose tissue softens the tissue and make it more stretchable, but this observation has so far no scientific basis. Another speculation could be that the perineal body is larger in obese women giving more distance between the vagina and rectum. This hypothesis was not supported in a recent study measuring perineal body length where there were no difference in maternal BMI in the group with perineal body length ≥3 cm compared to a group of women with a perineal body length of <3 cm [21]. Whether the thickness of the perineal body differs between obese and normal weight women could be a subject for future research.

Maternal obesity is associated with a number of severe and threatening obstetric and neonatal outcomes [5] which impacts the management of these women during pregnancy and delivery. The difference in absolute risk of having any anal sphincter laceration during delivery between normal weight women and morbidly obese women was 6.6 versus 6.0% and comparable figures for total anal sphincter injury was 1.3 versus 0.7%. This information could be clinically relevant to add when discussing the optimal way to deliver morbidly obese women.

The advantage of population-based register studies is the large number of individuals available for evaluation, which makes it possible to divide the study population into subgroups with sufficient numbers in each stratum and gives high statistical power. Sufficient number of study subjects made it possible to evaluate the three subgroups of obesity suggested by WHO, obesity classes I–III as well as three subgroups of anal sphincter injuries based on the degree of damage. Another advantage is the access to information available in the register on other related risk factors that could be confounders.

The drawback with register studies is obvious given the large size of the study and the numbers of health care units involved that the criteria for diagnosis** (**ICD codes**) **to define outcomes may not be uniform across the study population but the variation is probably not related to maternal BMI. Another shortcoming is that only variables included in the register could be either analysed or incorporated as putative confounders. The Swedish medical birth register has, for example, no information on women's ethnicity or on their socioeconomic status.

## 5. Conclusion

Maternal obesity in all three obesity classes seemed to decrease the risk for all three degrees of anal sphincter injuries after adjustment for instrumental delivery, birth weight, and late fetal head position. The strongest risk factor for anal sphincter laceration was high birth weight but, given equal size of the infant, the risk of anal sphincter injury decreased slightly with increasing maternal BMI. So based on these data, maternal obesity seems to be associated with less serious pelvic floor damages.

## Figures and Tables

**Table 1:** Risk factors for any anal sphincter injury among primiparous women with singleton vaginal cephalic births at term. OR for each variable studied in relation to anal sphincter injury was adjusted for all other variables and for year of delivery.

	Reference population (*n*)	Anal Sphincter injury (*n*)	%	Adjusted OR	95% CI
Maternal body mass index					
Unknown	50521	3351	6.6	0.96	0.93–1.00
Less than 18.5	12154	784	6.5	1.31	1.23–1.41
18.5–24.9	260037	17251	6.6	1.00	Reference
25–29.9	82638	5592	6.8	0.92	0.89–0.95
30–34.9	22733	1443	6.3	0.87	0.82–0.92
35–39.9	6362	371	5.8	0.77	0.69–0.85
40 or more	2037	123	6.0	0.75	0.65–0.90
Maternal age, y					
Younger than 20	18327	395	2.1	0.35	0.32–0.39
20–24	98749	4276	4.2	0.65	0.63–0.68
25–29	162898	11483	6.6	1.00	Reference
30–34	118037	9716	7.6	1.13	1.10–1.16
35–39	33528	2702	7.5	1.07	1.03–1.12
40–44	4803	337	6.6	0.91	0.81–1.02
45 or older	140	6	4.1	0.52	0.22–12.3
Fetal head position					
Occipitoanterior position	420006	27004	6.4	1.00	Reference
Occipitoposterior position	13955	1596	11.4	1.36	1.29–1.44
Other	2521	315	12.5	1.54	1.34–1.74
Instrumental delivery					
No	368519	18170	4.9	1.00	Reference
Yes	67963	10745	15.8	2.74	2.67–2.81
Infant birth weight					
Less than 3000	55550	1424	2.5	0.80	0.75–0.85
3000–3499	167059	7415	4.2	1.00	Reference
3500–3999	155742	11893	7.1	1.37	1.33–1.41
4000–4499	50373	6556	11.5	2.67	2.58–2.77
4500–4999	7117	1445	16.9	6.33	5.97–6.92
5000 or more	641	182	22.1	11.56	9.95–13.44

**Table 2:** Risk factors for partial anal sphincter injury (*N* = 19923), total anal sphincter injury (*N* = 5456), and total anal sphincter injury involving the anal/rectal mucosa (*N* = 2500) among primiparous women with singleton vaginal cephalic births at term. OR for each variable studied in relation to anal sphincter injury was adjusted for all other variables and for year of delivery.

	Partial anal sphincter injury ICD O70.2A%	Adjusted OR	95% CI	Total anal sphincter injury ICD O70.2B%	Adjusted OR	95% CI	Total anal sphincter injuries involving the anal/rectal mucosa ICD O70.3%	Adjusted OR	95% CI
Maternal body mass index									
Unknown	4.4	0.99	0.94–1.04	1.2	0.89	0.81–0.96	0.5	0.92	0.80–1.04
Less than 18.5	4.1	1.24	1.14–1.36	1.4	1.48	1.27–1.74	0.6	1.42	1.12–1.81
18.5–24.9	4.4	1.00	Reference	1.3	1.00	Reference	0.6	1.00	Reference
25–29.9	4.4	0.92	0.88–0.95	1.3	0.90	0.84–0.97	0.6	0.95	0.88–1.05
30–34.9	4.3	0.90	0.84–0.96	1.1	0.77	0.67–0.88	0.5	0.78	0.64–0.94
35–39.9	3.9	0.79	0.70–0.90	1.0	0.65	0.50–0.83	0.5	0.79	0.56–1.12
40 or more	4.2	0.82	0.66–1.02	0.7	0.47	0.28–0.78	0.7	0.84	0.48–1.44
Maternal age, y									
Younger than 20	1.3	0.32	0.28–0.36	0.5	0.46	0.38–0.57	0.2	0.44	0.32–0.59
20–24	2.8	0.64	0.61–0.68	0.9	0.68	0.63–0.74	0.4	0.67	0.60–0.75
25–29	4.6	1.00	Reference	1.3	1.00	Reference	0.6	1.00	Reference
30–34	5.5	1.16	1.13–1.20	1.5	1.07	1.00–1.14	0.7	1.01	0.92–1.11
35–39	5.5	1.14	1.09–1.21	1.4	0.96	0.86–1.06	0.6	0.84	0.71–0.98
40–44	5.1	1.02	0.89–1.16	1.0	0.73	0.55–0.96	0.4	0.56	0.36–0.80
45 or older	3.4	1.74	1.18–1.36	0.7	—	—	—	—	—
Fetal head position									
Occipitoanterior position	4.3	1.00	Reference	1.2	1.00	Reference	0.6	1.00	Reference
Occipitoposterior position	6.8	1.27	1.18–1.36	2.5	1.60	1.43–1.79	1.1	1.56	1.32–1.84
Others	7.6	1.49	1.28–1.32	2.5	1.66	1.21–2.12	0.9	1.25	0.83–1.89
Instrumental delivery									
No	3.3	1.00	Reference	0.9	1.00	Reference	0.4	1.00	Reference
Yes	9.5	2.62	2.58–2.70	2.0	2.93	2.78–3.10	1.4	2.92	2.70–3.16
Infant birth weight									
Less than 3000	1.8	0.80	0.75–0.86	0.4	0.80	0.64–0.92	0.2	0.74	0.58–0.94
3000–3499	3.1	1.00	Reference	0.8	1.00	Reference	0.3	1.00	Reference
3500–3999	5.0	1.32	1.28–1.37	1.4	1.43	1.34–1.53	0.7	1.50	1.36–1.65
4000–4499	7.8	2.52	2.42–2.63	2.6	2.88	2.67–3.10	1.3	3.20	2.87–3.56
4500–4999	11.4	5.60	5.21–6.03	4.2	7.91	6.9–8.93	2.3	9.77	8.26–11.54
5000 or more	14.2	8.99	7.40–10.91	5.9	16.05	12.06–21.37	2.9	21.54	13.91–33.38

**Table 3:** The effect of fetal head position at delivery and the effect of instrumental delivery on the risk for any anal sphincter injury according to maternal body mass index among primiparous women with singleton vaginal cephalic births at term.

Maternal BMI	Occiput posterior		Occiput anterior		OR*	95% CI	Instrumental vaginal delivery		Spontaneous vaginal delivery		OR**	95% CI
	No anal sphincter injury (*n*)	Anal sphincter injury (*n*)	No anal sphincter injury (*n*)	Anal sphincter injury (*n*)			No anal sphincter injury (*n*)	Anal sphincter injury (*n*)	No anal sphincter injury (*n*)	Anal sphincter injury (*n*)		
Unknown	1583	3140	48720	3140	1.23	1.04–1.46	2106	1245	42601	2106	2.70	2.50–2.91
Less than 18.5	361	32	11775	743	1.21	0.81–1.80	482	302	10410	482	3.12	2.68–3.14
18.5–24.9	8037	959	250460	16099	1.44	1.34–1.55	10949	6302	220189	10949	2.71	2.62–2.80
25–29.9	2972	213	79157	5221	1.19	1.05–1.35	3419	2123	68964	3419	2.78	2.62–2.94
30–34.9	774	96	21853	1336	1.62	1.28–2.05	875	568	19208	875	3.15	2.81–3.54
35–39.9	210	19	6165	351	1.27	0.77–2.09	259	112	5367	259	2.02	1.58–2.58
40 or more	70	0	1954	114	—	—	80	43	1700	80	2.21	1.46–3.06

*Adjustments were made for year of birth, maternal age, birth weight, and instrumental delivery. **Adjustments were made for year of birth, maternal age, birth weight, and fetal head position.
